# Declined Circulation and Seasonal Shifts of Human Coronavirus 229E in the Republic of Korea: Implications for Respiratory Virus Surveillance

**DOI:** 10.3390/pathogens15020231

**Published:** 2026-02-19

**Authors:** Mi-Ru Oh, Jeong Su Han, Sung Hun Jang, Ga-Yeon Kim, Jae Kyung Kim

**Affiliations:** 1 Department of Biomedical Laboratory Science, College of Health Sciences, Dankook University, Cheonan-si 31116, Chungnam, Republic of Korea; mrmr1028@naver.com (M.-R.O.); jshan1162@naver.com (J.S.H.); 2 Department of Medical Laser, Graduate School of Medicine, Dankook University, Cheonan-si 31116, Chungnam, Republic of Korea; well8143@naver.com; 3 Research Center for Bio-Functional and Biocompatible Materials, Dankook University, Cheonan-si 31116, Chungnam, Republic of Korea; 4 Department of Public Health, Graduate School, Dankook University, Cheonan-si 330714, Chungnam, Republic of Korea; sysnhj77@gmail.com

**Keywords:** age-specific susceptibility, human coronavirus 229E (HCoV-229E), respiratory virus surveillance, seasonality, Sustainable Development Goal 3 (SDG 3)

## Abstract

Human coronavirus 229E (HCoV-229E) is an alphacoronavirus that typically causes mild upper respiratory infections but remains understudied in terms of its long-term immuno-ecological behavior. Although the COVID-19 pandemic markedly altered human behavior and viral transmission, extended circulation patterns of HCoV-229E remain poorly defined. We analyzed annual, seasonal, and age-specific trends using real-time PCR–based respiratory virus surveillance data from Dankook University Hospital collected between 2007 and 2024. Among 23,284 nasopharyngeal swab specimens, 344 were positive for HCoV-229E (overall positivity, 1.43%). Positivity declined significantly over time (OR per year, 0.916; 95% CI, 0.894–0.939; *p* < 0.001). Compared with spring (1.04%), positivity was highest in winter (2.69%) and lowest in summer (0.29%) (both *p* < 0.001), whereas autumn (0.81%) showed no significant difference. Early childhood (1–5 years) demonstrated a higher likelihood of positivity than infants aged 0 years (aOR, 1.51; *p* = 0.007) and the highest crude positivity rate (1.89%). Although underlying mechanisms were not directly assessed, this long-term analysis documents a persistent decline and attenuation of seasonal dominance in HCoV-229E detection beyond the period of pandemic-related suppression. These findings underscore the value of sustained laboratory-based surveillance in identifying and tracking long-term changes in respiratory virus circulation patterns and in supporting public health monitoring aligned with Sustainable Development Goal 3 (SDG 3).

## 1. Introduction

Human coronavirus 229E (HCoV-229E) was first described in 1966 from respiratory specimens collected during surveillance of upper respiratory illness (winter 1962) and subsequently characterized in cell culture [1]. It has traditionally exhibited a stable winter-dominant seasonal pattern and has been recognized as a persistent component of the respiratory virus ecosystem [2]. However, recent surveillance data indicate a marked decline in detection frequency, suggesting that this trend may be partly attributable to shifts in the ecological balance of respiratory viruses. Nevertheless, methodological factors, including potential changes in diagnostic sensitivity, cannot be fully excluded [3].

HCoV-229E (Human coronavirus 229E) is a single-stranded positive-sense RNA (+ssRNA) virus belonging to the genus Alphacoronavirus [4]. Infection is initiated through the interaction of its envelope-associated spike glycoprotein (S) with the host aminopeptidase N (APN, CD13) receptor [5]. This molecular architecture supports preferential tropism for upper respiratory epithelial cells, consistent with the predominantly upper-respiratory clinical presentations reported for HCoV-229E infections [6]. Nonetheless, in immunocompromised individuals or older adults, HCoV-229E infection may progress to lower respiratory tract disease, pneumonia, or even acute respiratory distress syndrome (ARDS) [7]. Of the seven coronaviruses known to infect humans, HCoV-229E and HCoV-NL63 belong to the Alphacoronavirus genus, whereas HCoV-OC43, HCoV-HKU1, SARS-CoV, MERS-CoV, and SARS-CoV-2 are classified under Betacoronavirus [8]. Alphacoronaviruses possess relatively stable genomic structures and slower evolutionary rates, features that may influence long-term circulation dynamics [9].

Previous studies indicate that HCoV-229E circulates predominantly in winter and occurs more frequently in children than in adults [10]. However, many studies have relied on relatively short-term, pre-pandemic surveillance in specific settings, and often did not include detailed stratification by age and sex; consequently, systematic pre-pandemic versus post-pandemic comparisons remain limited [11]. Historically, HCoV-229E has received limited attention due to its low positivity rate (typically 1–2%) and predominantly mild clinical presentation, resulting in a scarcity of long-term surveillance data. Consequently, little is known about how its circulation patterns or seasonality have evolved over extended periods. Emerging evidence demonstrates that HCoV-229E circulation declined sharply during the COVID-19 pandemic and has not fully returned to pre-pandemic levels even after relaxation of public-health restrictions [12]. This sustained decline has been observed across multiple settings and cannot be fully explained by transient pandemic-related suppression alone. The COVID-19 pandemic profoundly altered human behavior, population-level immunity, and viral transmission structures. Non-pharmaceutical interventions (NPIs)—including masking, school closures, and social distancing—substantially disrupted the circulation of multiple respiratory viruses, including HCoV-229E. Yet the degree to which these ecological perturbations have resolved, and whether recovery trajectories differ by region or population group, remains poorly understood.

Cross-reactive immune responses between SARS-CoV-2 and endemic human coronaviruses, including HCoV-229E, have been reported, raising the possibility of immunological interactions between these viruses. Several studies suggest that humoral and cellular immune responses induced by SARS-CoV-2 infection may modulate reactivity to HCoV-229E, and conversely, prior exposure to alphacoronaviruses may influence susceptibility to SARS-CoV-2 [13]. Such immunological interactions may contribute to inter-coronavirus competition, host immune memory, and the factors influencing seasonal re-emergence. Thus, the persistent reduction and incomplete rebound of HCoV-229E circulation may reflect not only pandemic-era suppression but also immunologic imprinting and ecological replacement driven by widespread SARS-CoV-2 exposure. These possibilities should be regarded as immuno-ecological hypotheses rather than confirmed mechanisms, as our dataset does not directly assess cross-reactivity or viral competition.

Given this context, rather than emphasizing its clinical burden, HCoV-229E may serve as a descriptive case for examining how a low-prevalence endemic coronavirus behaves over extended surveillance periods. Subtle changes in its circulation and seasonal amplitude may serve as early markers of broader ecosystem-level shifts. Quantifying long-term patterns using accumulated molecular diagnostic data can provide valuable insight into pandemic-associated structural changes in viral circulation and host adaptation dynamics.

Therefore, the present study analyzed 18 years (2007–2024) of real-time PCR respiratory virus surveillance data from Dankook University Hospital to characterize the annual, seasonal, and age-specific patterns of HCoV-229E infection. Given the low endemic prevalence of HCoV-229E, this study focuses on aggregated temporal and seasonal detection patterns rather than detailed subgroup effect-size estimation. We evaluated long-term positivity trends, the weakening of seasonal amplitude, higher susceptibility observed in early childhood (1–5 years), and distinct pre- and post-pandemic circulation patterns. These findings support the role of long-term laboratory-based surveillance in contextualizing post-pandemic respiratory virus trends relevant to public health monitoring, including efforts aligned with Sustainable Development Goal 3.

## 2. Materials and Methods

### 2.1. Research Design

This retrospective observational study analyzed the detection results of HCoV-229E from 23,284 nasopharyngeal swab specimens collected between January 2007 and December 2024 at Dankook University Hospital, a tertiary medical center in Cheonan, Republic of Korea (Figure S2). During the study period, hospitalized and outpatient individuals with respiratory symptoms (such as sputum production, cough, or dyspnea) were included based on the clinical judgment of physicians. No major institutional restrictions or policy-level changes regarding the availability of multiplex PCR testing were implemented during the study period. However, variations in physician ordering practices and patient composition over time cannot be entirely excluded. Records with missing essential demographic information (age, sex, or test date) were excluded to maintain data integrity. Inconclusive test results were also removed to ensure data quality.

### 2.2. Laboratory Testing

Collected specimens were tested immediately after sampling; if immediate testing was not possible, they were stored in a 4 °C specimen storage room and analyzed within 24 h. Viral RNA was extracted using the QIAamp Viral RNA Mini Kit (QIAGEN, Hilden, Germany). From 2007 to 2012, respiratory viruses were detected using the Seeplex RV series multiplex PCR assays (Seegene, Seoul, Republic of Korea), which employed conventional PCR followed by gel electrophoresis. Since 2013, the laboratory has used the AdvanSure RV and RV-Plus real-time RT-PCR kits (LG Chem, Seoul, Republic of Korea), with amplification performed on the SLAN Real-Time PCR System (LG Chem, Seoul, Republic of Korea). All assays were conducted according to the manufacturer’s standard protocols and internal quality control guidelines. The AdvanSure RV kit targets fourteen major respiratory pathogens, including human coronavirus 229E, confirming that 229E detection has been consistently available since 2013. The Seeplex RV assay used before 2013 also included HCoV-229E as a target, ensuring continuity of 229E detection throughout the entire study period. All positive HCoV-229E results obtained between 2007 and 2024 were included in this study, with acknowledgement that the earlier data (2007–2012) were generated using different multiplex PCR methods.

### 2.3. Variable Definition and Seasonal Classification

Patient age was categorized according to the age classifications commonly used in clinical and epidemiological research as follows: infants (0 years), early childhood (1–5 years), kindergarten age (6–8 years), elementary school age (9–12 years), adolescents (13–18 years), adults (19–64 years), and older adults (≥65 years). This classification aligns with widely accepted criteria in clinical and epidemiological studies and was applied consistently throughout this research. In addition, the data were stratified by year, sex, and season. Seasons were defined as spring (March–May), summer (June–August), autumn (September–November), and winter (December–February). The variables analyzed in this study were limited to demographic and test-related characteristics, including age, sex, season, and year.

### 2.4. Data Analysis

Statistical analyses were performed using R software version 4.3.3 ( R Foundation for Statistical Computing, Vienna, Austria). Annual trends in HCoV-229E positivity were evaluated using a binomial logistic regression model to assess long-term changes over time [14,15]. For annual trends, we modeled year-level aggregated counts as a binomial response (positive vs. negative specimens per year), with calendar year as a continuous predictor. Seasonal variation was analyzed using logistic regression adjusted for year to account for temporal confounding effects. In the seasonal regression, spring was used as the reference category as a transitional season with intermediate activity, enabling straightforward interpretation of contrasts with the peak season (winter) and the low-activity season (summer); the choice of reference does not affect model fit or statistical significance. Sex- and age-specific differences in HCoV-229E positivity were also examined using logistic regression models. Odds ratios (ORs) and 95% confidence intervals (CIs) were derived from the model coefficients using Wald-type intervals: OR = exp(β) and 95% CI = exp (β ± 1.96 × SE), where SE is the standard error of β from the fitted model. Model diagnostics included evaluation of overdispersion by examining the ratio of residual deviance to degrees of freedom. When overdispersion was identified, quasibinomial logistic regression models were fitted as sensitivity analyses. In addition, age-specific positivity rates were compared using Pearson’s chi-square test of independence to assess the association between age group and HCoV-229E positivity [16].

These methods are widely accepted for epidemiologic surveillance data with binary (positive/negative) outcomes because they enable covariate-adjusted estimation of temporal and subgroup differences using an established effect measure (odds ratio) with standard uncertainty quantification. In addition, evaluating overdispersion and applying quasibinomial sensitivity analyses are commonly used to ensure robust inference when extra-binomial variation is present.

### 2.5. Ethical Considerations

This study was conducted in compliance with the ethical principles of the Declaration of Helsinki and was approved by the Institutional Review Board of Dankook University (IRB approval number: DKU 2025-02-004-003; approval date: 23 April 2025). As the research was retrospectively designed using de-identified data, the requirement for individual informed consent was waived.

## 3. Results

### 3.1. Demographic Characteristics of the Study Population

Between 2007 and 2024, a total of 23,284 nasopharyngeal swab specimens were tested for HCoV-229E. The median age of the tested population was 3 years (IQR, 0.82–55 years), with an overall age range of 0–100 years. Males accounted for 13,961 (60.0%) of the tested specimens, whereas females accounted for 9323 (40.0%). By age group, early childhood (1–5 years) accounted for the largest proportion of the tested population (38.16%), followed by older adults aged ≥ 65 years (20.15%), infants aged 0 years (19.51%), adults aged 19–64 years (12.61%), kindergarten-aged children 6–8 years (4.19%), elementary school–aged children 9–12 years (2.90%), and adolescents aged 13–18 years (2.48%) (Table 1).

### 3.2. Annual Trends

**Figure 1:**
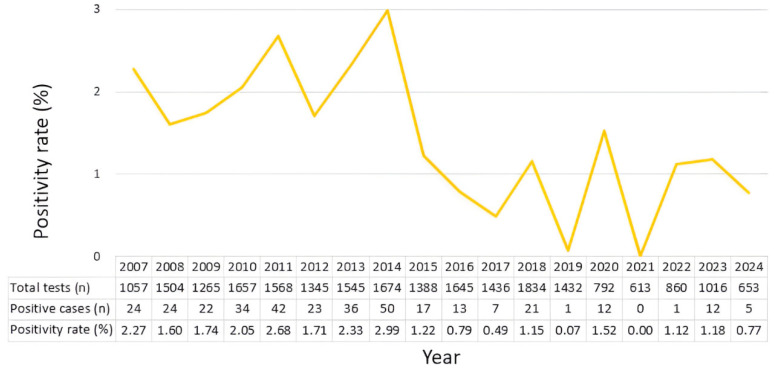
Annual positivity rates of HCoV-229E from 2007 to 2024. Each point represents the annual positivity rate (%) of HCoV-229E detected from 2007 to 2024. The table beneath the figure provides the corresponding raw annual data, including the number of tests performed, the number of positive cases, and the calculated positivity rate for each year.

A total of 23,284 nasopharyngeal swab specimens collected between 2007 and 2024 were analyzed, of which 344 tested positive for HCoV-229E, yielding an overall mean positivity rate of approximately 1.43%. Overall, the annual positivity rate declined across the study period; however, two transient peaks were observed in 2011 and 2014 (Table S1; Figure 1).

Binomial logistic regression analysis demonstrated a significant negative association between year and HCoV-229E positivity (regression coefficient = −0.087, standard error = 0.012, z = −7.059, *p* < 0.001). The model showed a Null deviance of 144.096 (df = 17) and a Residual deviance of 91.039 (df = 16), with an AIC value of 170.83, indicating an adequate model fit. The model converged stably after five Fisher scoring iterations. The odds ratio (OR) per one-year increase in year was 0.916 (95% CI: 0.894–0.939, *p* < 0.001), corresponding to a 5-year OR of 0.646 (95% CI: 0.572–0.729).

Model diagnostics for the annual logistic regression revealed substantial overdispersion (φ = 5.69). To address this, a quasibinomial logistic regression model was fitted as a sensitivity analysis. After adjusting for overdispersion, the standard errors increased, leading to a larger (more conservative) *p*-value, while the OR estimate remained unchanged. The quasibinomial model yielded effect estimates that were nearly identical to those of the standard binomial model (OR per year = 0.916), and the association between year and positivity remained statistically significant (*p* = 0.0067), supporting the robustness of the observed long-term decline.

### 3.3. Seasonal Patterns

The estimated positivity rates by season were 1.04% in spring, 0.29% in summer, 0.81% in autumn, and 2.69% in winter, indicating that winter had the highest positivity in absolute terms. After adjustment for year, season had a statistically significant effect on positivity. Compared with spring, winter showed a significantly higher odds ratio (OR, 2.62; 95% CI, 2.01–3.42; *p* < 0.001), whereas summer showed a significantly lower odds ratio (OR, 0.28; 95% CI, 0.16–0.48; *p* < 0.001). Autumn did not differ significantly (OR, 0.77; 95% CI, 0.55–1.10; *p* = 0.15); Table 2. Additional pairwise contrasts further confirmed that winter had significantly higher odds of positivity than both summer (OR = 9.44, *p* < 0.001) and autumn (OR = 3.39, *p* < 0.001).

A significant season-by-year interaction was observed (ΔDeviance = 14.04, df = 3, *p* = 0.0029), suggesting that the relative differences in positivity among seasons varied across years rather than remaining constant. Overall, winter showed the highest positivity; however, in some years—such as 2010 and 2014—autumn exceeded winter, and in recent years spring (2023) or summer (2024) showed higher positivity than winter (Table S2).

**Figure 2:**
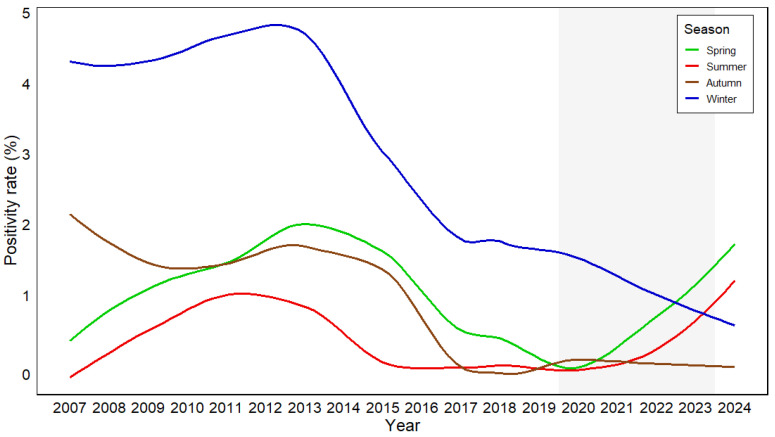
LOESS-Smoothed Seasonal Trends in HCoV-229E Positivity From 2007 to 2024. Season-specific LOESS-smoothed curves depicting long-term positivity trends of HCoV-229E across spring (green), summer (red), autumn (brown), and winter (blue). Shaded region indicates the COVID-19 pandemic period (2020–2023). Positivity rates are shown on the y-axis, and calendar years from 2007 to 2024 are displayed on the x-axis.

In a separate analysis restricted to winter data, the odds of positivity decreased gradually over the study period (OR per year, 0.91; *p* < 0.001), indicating a progressive downward trend in winter positivity by year (Figure 2).

### 3.4. Age- and Sex-Specific Differences in HCoV-229E Positivity

**Figure 3:**
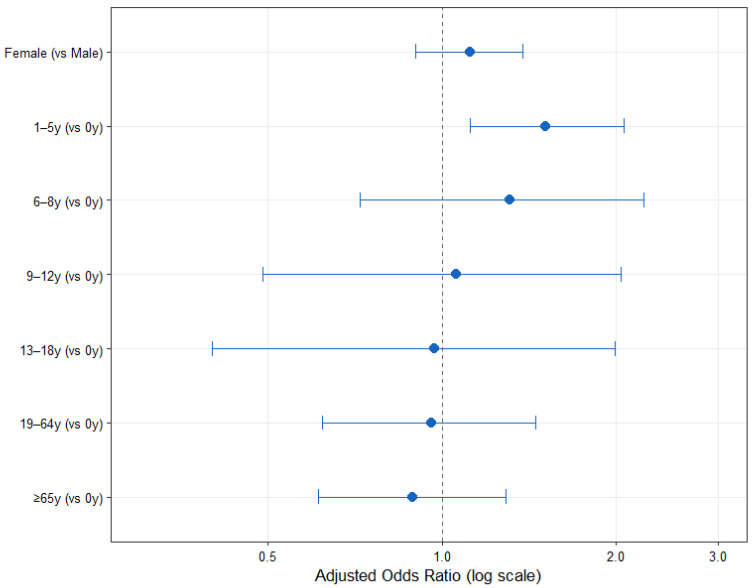
Adjusted odds ratios for HCoV-229E positivity by age and sex Reference groups: age 0 years and male. The x-axis is on a logarithmic scale.

Compared with infants aged 0 years, only early childhood (1–5 years) showed a significantly higher likelihood of being positive (aOR = 1.51, 95% CI: 1.12–2.06, *p* = 0.007). No significant differences were observed in other age groups—6–8 years (aOR = 1.31, *p* = 0.347), 9–12 years (aOR = 1.06, *p* = 0.872), 13–18 years (aOR = 0.97, *p* = 0.930), 19–64 years (aOR = 0.96, *p* = 0.832), and ≥65 years (aOR = 0.89, *p* = 0.535; Table 3). Sex was not a significant factor associated with HCoV-229E positivity, and no notable difference in positivity rates was observed between males and females (aOR = 1.12, 95% CI: 0.90–1.38, *p* = 0.319; Figure 3).

Comparison of age-specific positivity rates revealed a significant association between age group and HCoV-229E positivity (χ^2^ = 18.55, df = 6, *p* < 0.01). The positivity rate was highest in early childhood (1–5 years; 1.89%) and showed a gradual decline with increasing age (Table 4).

Each point represents the adjusted odds ratio (aOR) for HCoV-229E positivity, and the horizontal lines indicate the corresponding 95% confidence intervals (CIs). The vertical dashed line denotes an odds ratio of 1.0, representing the reference level. Values plotted to the right of the line indicate a higher likelihood compared with the reference group, while those to the left indicate a lower likelihood.

## 4. Discussion

This study analyzed 18 years of molecular surveillance data (2007–2024) and identified convergent patterns in the circulation of HCoV-229E, providing new insights into its temporal dynamics and ecological behavior in the post-pandemic era. The annual positivity rate showed a gradual decline, with a marked suppression during the pandemic period, particularly in 2021, followed by only partial recovery thereafter. In our study, the HCoV-229E positivity rate showed a long-term declining trend; however, two localized peaks were observed in 2011 and 2014. These transient increases are consistent with the interannual variability reported for seasonal human coronaviruses and suggest that multiple factors, rather than a single driver, may have contributed. Because long-term protective immunity may not be fully sustained after infection with seasonal human coronaviruses, fluctuations in population-level immunity (i.e., accumulation of susceptible individuals) combined with changes in the transmission environment could lead to year-to-year and multi-year variation in epidemic intensity [7]. In addition, antigenic evolution of HCoV-229E—particularly changes in the spike protein with potential antibody escape—has been reported and may provide a plausible background explanation for relatively higher detection in specific years [17]. Climatic conditions may also modulate transmission efficiency during winter, and the Republic of Korea hospital-based pediatric study reported a negative association between HCoV-229E detection and ambient air temperature [10]. Nevertheless, because this single-center retrospective analysis did not integrate year-specific meteorological data, genotype/lineage dynamics, co-circulating viruses, or changes in healthcare utilization and testing indications, the peaks in 2011 and 2014 cannot be attributed to any specific factor. Similar downward trends have been reported in surveillance studies from Japan, Germany, and Bulgaria [18,19,20]. However, uninterrupted national time series spanning the full 2007–2024 interval, comparable to the present study, remain scarce. Nevertheless, long-term hospital-based surveillance from coastal Kenya reported frequent co-detections among seasonal human coronaviruses and did not show a consistently well-defined seasonal pattern, highlighting the heterogeneity of endemic HCoV circulation across settings [21]. However, these studies were based on short-term or pandemic-restricted datasets and were therefore limited in their ability to assess long-term changes in detection patterns. In contrast, our uninterrupted 18-year time series allows the detection of long-range shifts that have not been previously documented. In particular, the statistically significant season–year interaction provides long-term evidence supporting a progressive attenuation of classical winter dominance, accompanied by persistently low post-pandemic activity through 2024 and distinct age-specific susceptibility patterns that could not be discerned in earlier national or multicenter reports.

This sustained reduction occurred alongside the inherently slow evolutionary rate of HCoV-229E and strong external epidemiological pressures imposed during the COVID-19 pandemic, including non-pharmaceutical interventions such as mask use, school closures, and social distancing [22,23,24]. Previous work has demonstrated that such measures not only suppressed HCoV-229E transmission but also significantly reduced circulation of other respiratory viruses such as RSV and influenza [25,26].

As an alphacoronavirus, HCoV-229E possesses a comparatively conserved genome with limited antigenic variability. Its receptor-binding domain (RBD) appears to induce weaker neutralizing antibody responses than those elicited by betacoronaviruses such as SARS-CoV-2 or MERS-CoV, suggesting a relatively constrained adaptive antigenic landscape [27]. The sustained decline and incomplete rebound observed in this study are consistent with a broader ecological displacement within the human coronavirus community, although alternative explanations, such as changes in healthcare-seeking or testing practices, cannot be entirely excluded. The emergence and global spread of SARS-CoV-2 may have altered the relative detection profiles of endemic human coronaviruses. This interpretation aligns with recent multinational reports showing persistently low post-pandemic activity of HCoV-229E [12]; notably, the lack of recovery between 2021 and 2024 cannot be readily explained by NPIs or environmental factors alone.

From an ecological perspective, the decline in HCoV-229E circulation may suggest a pattern that could be consistent with the combined influence of successive betacoronavirus introductions and potential cross-reactive immunity in human populations; however, these possibilities were not directly assessed in this study. Widespread exposure to SARS-CoV-2 could have contributed to humoral and cellular immune responses with partial cross-reactivity to HCoV-229E [28], although this possibility remains hypothetical and beyond the scope of the current dataset. Accordingly, any apparent shifts in HCoV-229E’s ecological position within the coronavirus community should be interpreted cautiously.

Understanding whether HCoV-229E will persist as an attenuated endemic virus, adapt through genetic drift, or continue to recede from clinical detection will require continued genomic surveillance. If the downward trend persists, routine testing for a pathogen with diminishing clinical relevance may become inefficient and necessitate reconsideration of diagnostic panel composition. Conversely, if HCoV-229E re-emerges, early molecular detection will be critical to determine whether resurgence reflects natural recrudescence or the appearance of novel variants through recombination or antigenic shifts. Thus, HCoV-229E surveillance serves not only as an indicator of seasonal coronavirus activity but also as a model for understanding ecological replacement and virus persistence in the post-pandemic era.

Seasonally, HCoV-229E retained its winter predominance, consistent with regional and international observations [29,30]. Higher positivity during cold, dry months may be linked to increased viral stability at low humidity, greater indoor crowding, and reduced mucociliary clearance [31,32,33]. However, long-term trends revealed a progressive weakening of this classical seasonal pattern. This indicates that the predominance of winter remained generally consistent throughout the study period, but the magnitude of that predominance fluctuated over time. Although winter generally showed the highest positivity, this pattern was not uniform across years: in some years (e.g., 2010 and 2014), autumn exceeded winter, and in the most recent period, spring (2023) or even summer (2024) showed higher positivity than winter. This attenuation of seasonal amplitude suggests that 229E transmission is no longer driven primarily by environmental factors such as temperature and humidity, but is increasingly shaped by immunologic forces and inter-viral ecological interactions, although this interpretation remains speculative in the absence of direct immune or genomic evidence. The erosion of seasonality may represent an early sign of “attenuation of classical seasonality” reflecting the transition of the human viral ecosystem toward a new post-pandemic equilibrium.

According to the age-stratified analyses, early childhood (1–5 years) exhibited the highest susceptibility, a pattern that was observed across complementary analytical approaches including age-based comparisons, and may reflect immunological immaturity and higher exposure in group settings. Infants also demonstrated relatively high positivity rates, potentially owing to waning maternal antibodies and limited innate and adaptive defenses [34,35]. In contrast, adults showed low infection rates, which can be explained by cumulative immunity acquired through repeated exposures. Antibody waning and seroreversion occur more rapidly in children than adults, and previous studies suggest that 20–39% of children experience annual reinfections, whereas adults exhibit slower antibody decay [36]. Overall, the risk of HCoV-229E infection was highest among early childhood (1–5 years), whereas no marked differences were found with advancing age. These findings support the idea that accumulated immune memory provides partial protection in older age groups, and the absence of sex differences indicates that susceptibility is primarily shaped by immunologic maturation rather than sex-specific factors. Additionally, widespread exposure to SARS-CoV-2 and vaccination may have further reduced HCoV-229E susceptibility in adults through cross-reactive immunity. Collectively, early childhood (1–5 years) may represent a key group contributing to alphacoronavirus circulation.

A major strength of this study is the use of an 18-year dataset spanning both pre- and post-pandemic periods, analyzed within a consistent diagnostic framework. By integrating ecological and immunological considerations—including shifts in betacoronavirus dominance, cross-immunity, and behavioral changes induced by NPIs—we provide a comprehensive interpretation of the long-term epidemiology of HCoV-229E. From a diagnostic perspective, our findings underscore the need to continually re-evaluate the clinical relevance of detected pathogens and to adapt molecular diagnostic panels in response to evolving epidemiologic conditions.

Beyond typical respiratory presentations, multiplex respiratory panels can also support differential diagnosis in mumps-like parotitis when mumps testing is negative, and may reveal concomitant viral detections. A recent case report described unilateral parotitis with concurrent detection of HCoV-OC43 and influenza A/H3N2, underscoring the practical value of laboratory testing for recognizing non-mumps viral etiologies and potential co-detections [37].

In other settings, post-pandemic rebounds may increase pediatric co-infections due to an immunity gap after prolonged suppression of routine viruses and atypical seasonal overlap during the rebound period, particularly in high-contact school settings and with broader multiplex PCR testing. Because co-infections were not directly assessed in our dataset, this interpretation should be considered hypothesis-generating.

Another notable observation is the asymmetric recovery of respiratory viruses after the pandemic. While many viruses—including RSV, rhinovirus, parainfluenza viruses, and influenza—exhibited rapid resurgence between 2021 and 2023, HCoV-229E did not. This discrepancy may be consistent with coronavirus-specific immune imprinting, whereby immunity generated through SARS-CoV-2 infection or vaccination could potentially influence long-term susceptibility to alphacoronaviruses. However, these immunologic interactions remain theoretical, as the present study did not directly evaluate immune imprinting or cross-reactive antibody dynamics. Therefore, the continued suppression of HCoV-229E should be interpreted cautiously and may reflect broader ecological changes, rather than definitive evidence of immuno-ecological restructuring.

Some limitations warrant consideration. First, the relatively small number of HCoV-229E–positive cases (344 cases over an 18-year period) represent a major limitation of this study, reflecting the low endemic prevalence of this virus and potentially limiting statistical power for certain subgroup analyses; however, the extended duration of surveillance provides a rare opportunity to characterize long-term temporal and seasonal patterns of HCoV-229E circulation. Second, this single-center retrospective study may not fully represent national trends, as hospital-based testing can be influenced by healthcare-seeking behavior, outbreak response, and changes in specimen submission patterns over time. Third, clinical variables such as symptomatology, co-infections, and disease severity were unavailable, precluding assessment of the clinical spectrum and prognostic impact of HCoV-229E infection. Fourth, because analyses were conducted at the specimen level, this study did not estimate population-based incidence, and the observed detection patterns may not directly reflect community-level transmission dynamics. Fifth, the transition from Seeplex RV to AdvanSure RV real-time RT-PCR before 2013 may have introduced minor variability in analytic sensitivity, as differences in amplification chemistry and detection thresholds between platforms cannot be completely excluded. This variability could theoretically influence long-term positivity patterns, including part of the observed decline, although parallel validation data were not available to formally quantify such effects. Nonetheless, both assays consistently targeted HCoV-229E, and all results were interpreted according to standardized laboratory protocols, suggesting that any platform-related influence on the overall multi-year trend is likely limited. In addition, a supplementary figure comparing positivity rates before and after the 2013 platform transition (Figure S1) does not indicate any obvious abrupt change around the transition year, suggesting that while assay-related factors cannot be fully excluded, they are unlikely to be the sole explanation for the observed long-term decline. Sixth, because test ordering was based on physicians’ clinical judgment, and such judgment may have evolved over the 18-year period, shifts in testing behavior could have influenced which specimens were submitted and may have contributed to changes in observed positivity patterns. Although annual testing volume increased gradually, the persistent post-pandemic reduction in HCoV-229E activity suggests that the decline is unlikely to be fully explained solely by broader testing utilization. Moreover, the lack of integrated meteorological, genomic, co-circulation, and testing-indication data limits causal inference regarding the drivers of the observed temporal peaks and shifts. Finally, the absence of genomic sequencing data prevented evaluation of viral evolution, recombination events, or antigenic changes. Despite these limitations, the epidemiologic patterns captured here provide meaningful evidence of long-term ecological changes in HCoV-229E circulation, including viral competition, immune restructuring, and early indications of seasonal attenuation.

In conclusion, the sustained decline, weakened seasonality, and persistent age-specific vulnerability observed in this study suggest an immuno-ecological reorganization within the human coronavirus community. These findings imply a shift in competitive coexistence and the emergence of a new ecological hierarchy in which alphacoronaviruses gradually lose their niche while betacoronaviruses—particularly SARS-CoV-2—gain dominance. Given its low positivity rate and potential susceptibility to cross-reactive immunity from other coronaviruses, HCoV-229E is unlikely to function as a definitive sentinel virus. However, its pronounced sensitivity to ecological perturbations suggests that it may still provide complementary insights into broader shifts within the respiratory viral ecosystem. Because its low detection frequency makes it highly sensitive to ecological perturbation, HCoV-229E can function as a practical model for transitioning from traditional “epidemic surveillance” toward “ecological surveillance.” Future research incorporating standardized multi-center genomic monitoring will be essential to clarify the post-pandemic evolutionary trajectory of HCoV-229E and to develop adaptive surveillance and diagnostic strategies that integrate viral genetics, human behavioral interventions, and ecological competition. Together, these findings support improved understanding of long-term respiratory virus dynamics and may contribute to public health surveillance strategies aligned with Sustainable Development Goal 3, thereby helping to refine infectious-disease monitoring and strengthen public-health preparedness in the post-pandemic era.

## 5. Conclusions

This long-term analysis demonstrated a gradual decline in HCoV-229E positivity, accompanied by a stepwise attenuation of winter predominance. Positivity was highest in early childhood (1–5 years), whereas no significant sex-related differences were observed. Although the underlying mechanisms could not be directly evaluated using this single-center, PCR-based dataset, the present study provides a descriptive account of long-term changes in HCoV-229E detection patterns across the pre- and post-pandemic periods. Given the low overall positivity rate, HCoV-229E may have limited utility as a stand-alone primary surveillance indicator; however, continued molecular monitoring may help interpret and contextualize post-pandemic trends in seasonal coronavirus detection.

## Figures and Tables

**Table 1:** Demographic characteristics of specimens tested for HCoV-229E (2007–2024).

Variable	Category	n	%, Median (IQR)
Total specimens tested		23,284	
Positive specimens		344	1.43%
Age (years)	Median (IQR)		3 (0.82–55)
	Range		0–100
Sex	Male	13,961	60.00%
	Female	9323	40.00%
	Total	23,284	100%
Age group	Infants (0 years)	4543	19.51%
	Early childhood (1–5 years)	8886	38.16%
	Kindergarten age (6–8 years)	976	4.19%
	Elementary school age (9–12 years)	675	2.90%
	Adolescents (13–18 years)	577	2.48%
	Adults (19–64 years)	2935	12.61%
	Older adults (≥65 years)	4692	20.15%
	Total	23,284	100%

**Table 2:** Logistic regression analysis of seasonal effects on 229E positivity.

Variable	Category	OR	95% CI	*p*-Value
Season	Spring (ref.)	1	–	–
	Summer	0.28	0.16–0.48	< 0.001
	Autumn	0.77	0.55–1.10	0.150
	Winter	2.62	2.01–3.42	< 0.001
Year	Continuous	0.91	0.90–0.94	<0.001
Interaction	Season × Year	–	–	0.0029

OR, odds ratio; CI, confidence interval.

**Table 3:** Factors associated with HCoV-229E positivity by age and sex (logistic regression analysis).

Variable	Category	aOR (Adjusted Odds Ratio)	95% Confidence Interval (CI)	*p*-Value
Sex	Male (reference)	1	–	–
	Female	1.12	0.90–1.38	0.319
Age group	0 years (reference)	1	–	–
	1–5 years	1.51	1.12–2.06	0.007
	6–8 years	1.31	0.72–2.23	0.347
	9–12 years	1.06	0.49–2.04	0.872
	13–18 years	0.97	0.40–1.99	0.930
	19–64 years	0.96	0.62–1.45	0.832
	≥65 years	0.89	0.61–1.29	0.535

**Table 4:** Age-specific positivity rates (%) of HCoV-229E according to tested population (2007–2024).

Age Group	Total Individuals (n)	Positive Cases (n)	Positivity Rate (%)
Infants (0 years)	4543	57	1.25
Early childhood (1–5 years)	8886	168	1.89
Kindergarten age (6–8 years)	976	16	1.63
Elementary school age (9–12 years)	675	9	1.33
Adolescents (13–18 years)	577	7	1.21
Adults (19–64 years)	2935	35	1.19
Older adults (≥65 years)	4692	52	1.10

Positivity rate was calculated as the number of HCoV-229E–positive cases divided by the total number of tested individuals in each age group.

## Data Availability

The dataset analyzed in this study was derived from laboratory records at Dankook University Hospital and is subject to institutional as well as national ethical guidelines. Due to confidentiality requirements and data protection regulations, the original raw data cannot be made publicly available. However, de-identified aggregated data may be provided by the corresponding author upon reasonable request and contingent on approval from the Institutional Review Board. All data access inquiries should be directed to the corresponding author.

## Supplementary Materials

The following supporting information can be downloaded at: https://www.mdpi.com/article/10.3390/pathogens15020231/s1, Table S1: Annual number of tests, positive and negative cases, and positivity rate of HCoV-229E detected between 2007 and 2024. Table S2: Number of respiratory specimens tested and positive HCoV-229E cases for each season in every year (2007–2024). Figure S1: Annual positivity rates of HCoV-229E 2007–2024, comparing the periods before and after the 2013 diagnostic platform transition. Figure S2. Study site location in the Republic of Korea: Cheonan-si highlighted.

## Abbreviations

The following abbreviations are used in this manuscript:

SDG	Sustainable Development Goals
HCoV-229E	Human coronavirus 229E
RSV	Respiratory Syncytial Virus
MERS-CoV	Middle East Respiratory Syndrome Coronavirus
NPI(s)	Non-pharmaceutical interventions
