# Supplementary material for: Declined Circulation and Seasonal Shifts of Human Coronavirus 229E in the Republic of Korea: Implications for Respiratory Virus Surveillance
Source: Pathogens. 2026 Feb 19;15(2):231. doi: 10.3390/pathogens15020231 (PMC12943526; doi:10.3390/pathogens15020231)
Supplement: Supplementary file 1 [file pathogens-15-00231-s001.zip › 229E Figure S2.pdf]

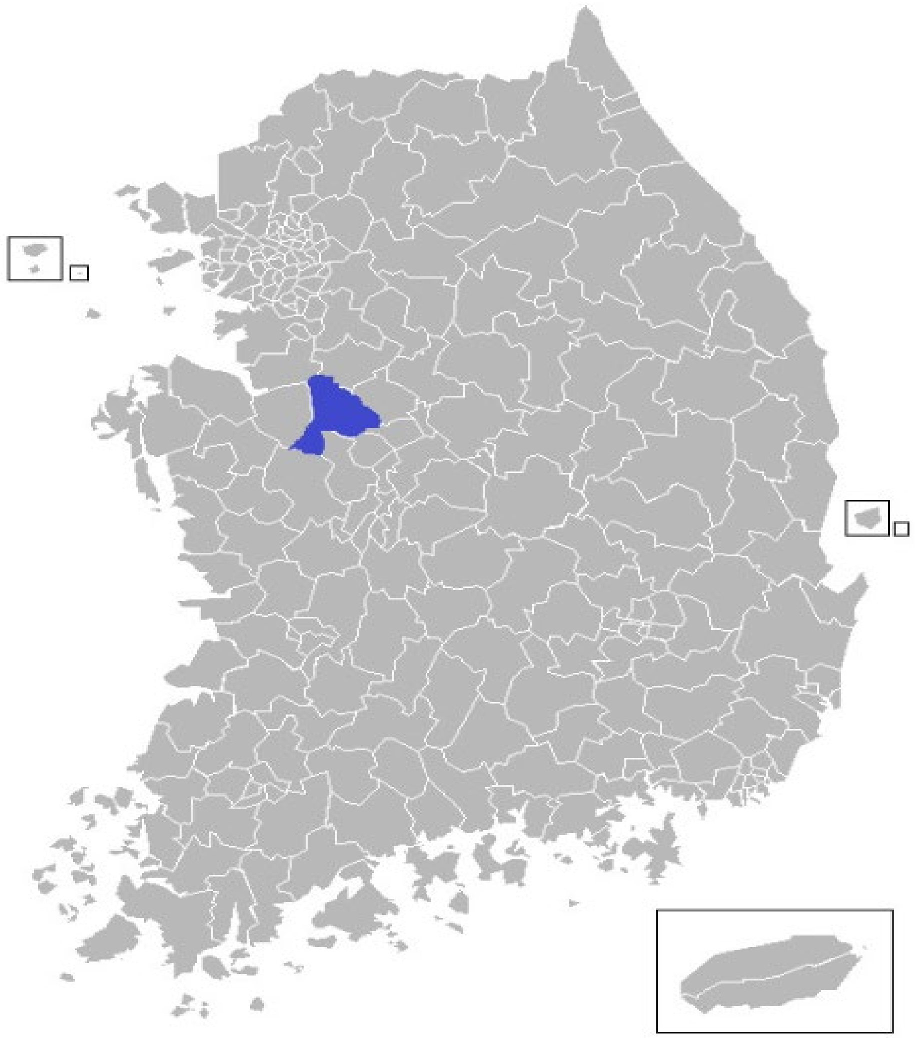

Figure S2. Study site location in the Republic of Korea: Cheonan-si highlighted.

Cheonan-si (study site) is highlighted in blue on an administrative boundary map of the Republic of Korea.

Base map: *"Administrative divisions map of Republic of Korea.svg"* by Dmthoth**,** Wikimedia Commons, licensed under CC BY-SA 3.0**.** Modified by the authors (Cheonan-si highlighted).
