# Supplementary material for: Declined Circulation and Seasonal Shifts of Human Coronavirus 229E in the Republic of Korea: Implications for Respiratory Virus Surveillance
Source: Pathogens. 2026 Feb 19;15(2):231. doi: 10.3390/pathogens15020231 (PMC12943526; doi:10.3390/pathogens15020231)
Supplement: Supplementary file 1 [file pathogens-15-00231-s001.zip › 229E_Figure_S1.pdf]

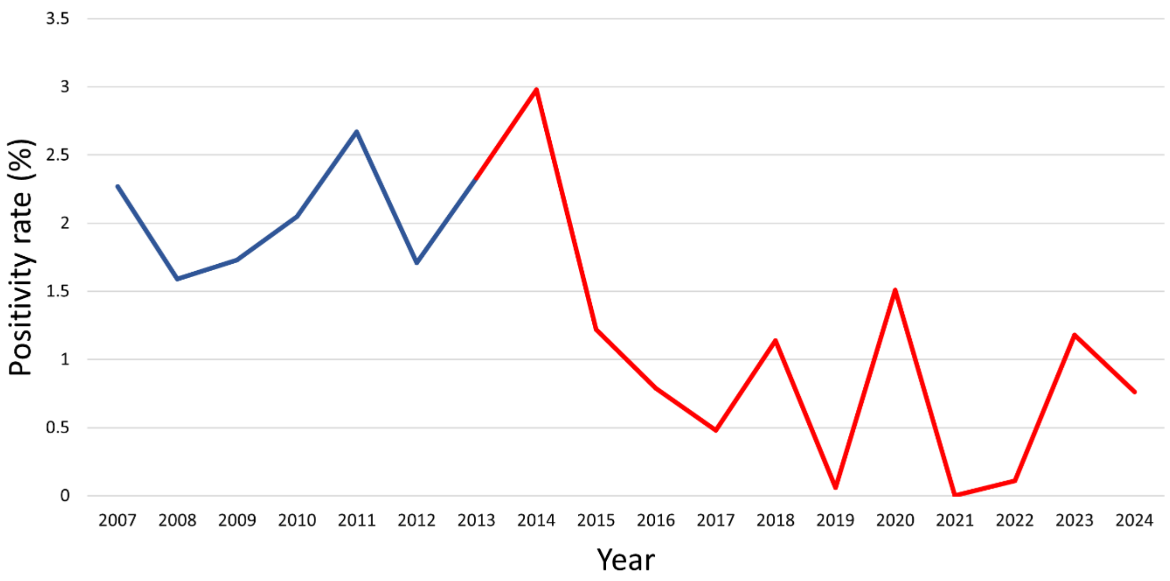

Figure S1. Annual positivity rates of HCoV-229E from 2007 to 2024, stratified by diagnostic platform before vs. after the 2013 transition.

Blue line (2007–2012): Seeplex RV assay; red line (2013–2024): AdvanSure RV real-time RT-PCR platform. Positivity rate was calculated as (annual positive cases / annual tests) × 100.
