# Supplementary material for: Declined Circulation and Seasonal Shifts of Human Coronavirus 229E in the Republic of Korea: Implications for Respiratory Virus Surveillance
Source: Pathogens. 2026 Feb 19;15(2):231. doi: 10.3390/pathogens15020231 (PMC12943526; doi:10.3390/pathogens15020231)
Supplement: Supplementary file 1 [file pathogens-15-00231-s001.zip › 229E_Table_S1.pdf]

Table S1. Annual number of tests, positive and negative cases, and positivity rate of HCoV-229E detected between 2007 and 2024.

Year	Total tests (n=23,284)	Positive cases (n=344)	Negative cases (n=22,940)	Positivity rate (%)
2007	1,057	24	1,033	2.27
2008	1,504	24	1,480	1.60
2009	1,265	22	1,243	1.74
2010	1,657	34	1,623	2.05
2011	1,568	42	1,526	2.68
2012	1,345	23	1,322	1.71
2013	1,545	36	1,509	2.33
2014	1,674	50	1,624	2.99
2015	1,388	17	1,371	1.22
2016	1,645	13	1,632	0.79
2017	1,436	7	1,429	0.49
2018	1,834	21	1,813	1.15
2019	1,432	1	1,431	0.07
2020	792	12	780	1.52
2021	613	0	613	0.00
2022	860	1	859	0.12
2023	1,016	12	1,004	1.18
2024	653	5	648	0.77
