# Supplementary material for: Declined Circulation and Seasonal Shifts of Human Coronavirus 229E in the Republic of Korea: Implications for Respiratory Virus Surveillance
Source: Pathogens. 2026 Feb 19;15(2):231. doi: 10.3390/pathogens15020231 (PMC12943526; doi:10.3390/pathogens15020231)
Supplement: Supplementary file 1 [file pathogens-15-00231-s001.zip › 229E_Table_S2.pdf]

Table S2. Seasonal numbers of respiratory specimens tested, HCoV-229E–positive cases, and positivity rates (%) by year (2007–2024).

Year	Season	Tests (n)	Positive cases (n)	Positivity rate (%)
2007	spring	256	0	0.00
2007	summer	176	0	0.00
2007	autumn	301	9	2.99
2007	winter	324	15	4.63
2008	spring	430	3	0.70
2008	summer	251	0	0.00
2008	autumn	397	2	0.5
2008	winter	426	19	4.46
2009	spring	354	8	2.26
2009	summer	208	1	0.48
2009	autumn	363	1	0.27
2009	winter	340	12	3.53
2010	spring	473	8	1.69
2010	summer	352	2	0.57
2010	autumn	393	14	3.56
2010	winter	439	10	2.28
2011	spring	434	3	0.69
2011	summer	279	1	0.36
2011	autumn	455	7	1.54
2011	winter	400	31	7.75
2012	spring	460	2	0.43
2012	summer	237	5	2.11
2012	autumn	278	2	0.71
2012	winter	370	14	3.78
2013	spring	518	19	3.67
2013	summer	372	3	0.81
2013	autumn	344	0	0.00
2013	winter	311	14	4.50
2014	spring	546	13	2.38
2014	summer	316	0	0.00
2014	autumn	322	16	4.97
2014	winter	490	21	4.29
2015	spring	426	5	1.17
2015	summer	249	0	0.00
2015	autumn	319	1	0.31
2015	winter	394	11	2.79
2016	spring	444	1	0.23
2016	summer	354	0	0.00
2016	autumn	378	0	0.00
2016	winter	469	12	2.56
2017	spring	343	3	0.87
2017	summer	285	0	0.00
2017	autumn	413	0	0.00
2017	winter	395	4	1.01
2018	spring	409	3	0.73
2018	summer	414	0	0.00

2018	autumn	367	0	0.00
2018	winter	644	18	2.80
2019	spring	391	0	0.00
2019	summer	357	0	0.00
2019	autumn	322	1	0.31
2019	winter	362	0	0.00
2020	spring	167	0	0.00
2020	summer	143	0	0.00
2020	autumn	178	0	0.00
2020	winter	304	12	3.95
2021	spring	131	0	0.00
2021	summer	129	0	0.00
2021	autumn	214	0	0.00
2021	winter	139	0	0.00
2022	spring	184	0	0.00
2022	summer	267	0	0.00
2022	autumn	237	0	0.00
2022	winter	172	1	0.58
2023	spring	270	9	3.33
2023	summer	284	1	0.35
2023	autumn	233	0	0.00
2023	winter	229	2	0.87
2024	spring	155	1	0.65
2024	summer	137	2	1.46
2024	autumn	93	0	0.00
2024	winter	268	2	0.75

Seasons were defined by specimen collection month (Spring: Mar–May; Summer: Jun–Aug; Autumn: Sep–Nov; Winter: Dec–Feb). For year-wise aggregation, 'Winter' refers to tests collected in Dec of the same calendar year and Jan–Feb of the same calendar year (calendar-year assignment).
